# The extent of pulmonary vein electrical connection and sex are independent predictors of the mechanistic role of pulmonary veins in persistent atrial fibrillation

**DOI:** 10.1007/s10840-025-02231-4

**Published:** 2026-01-12

**Authors:** Matteo Marchetti, Christelle Haddad, Adrian Luca, Mathieu Le Bloa, Cheryl Teres, Ciro Ascione, Mattia Pagnoni, Giulia Domenichini, Etienne Pruvot, Patrizio Pascale

**Affiliations:** 1 Arrhythmia Unit, Cardiovascular Department, Centre Hospitalier Universitaire Vaudois and University of Lausanne, Lausanne, 1011 Switzerland; 2 Arrhythmia Unit, Louis Pradel Cardiovascular Hospital, Hospices Civils de Lyon, Lyon, France

## Abstract

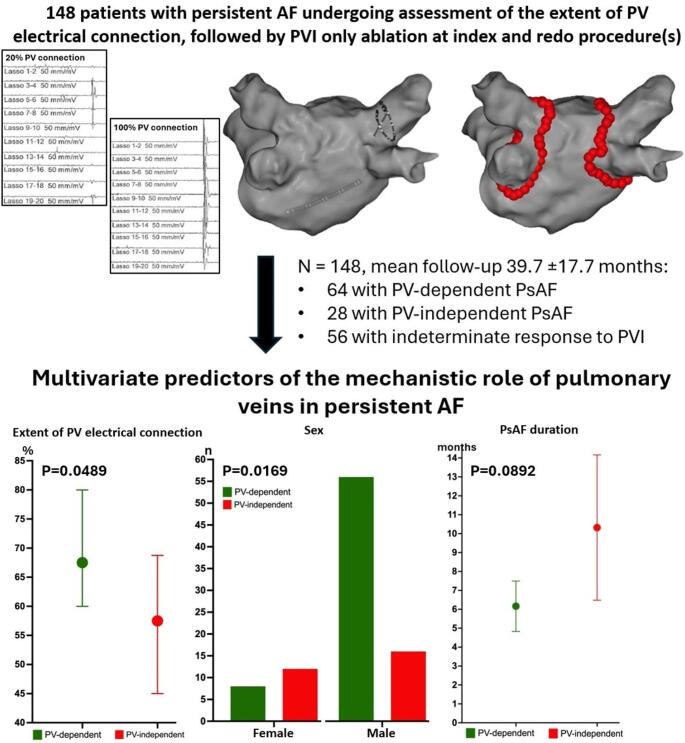

The lower success rate of pulmonary vein isolation (PVI) in persistent atrial fibrillation (PsAF) compared to paroxysmal AF reflects the fact that the mechanisms of PsAF may be independent of the PVs in a significant subset of patients. Distinguishing patients with PV-dependent from those with PV-independent PsAF could allow more efficient patient-tailored ablation strategies. We recently reported that the circumferential extent of PV electrical connection assessed during pre-ablation mapping (Fig. 1A) is correlated to the success rate of stand-alone PVI in patients with PsAF [1]. This study aimed to assess the relationship between the extent of PV connection and clinical variables to the likely mechanism of arrhythmogenesis by comparing patients with presumed PV-dependent versus PV-independent PsAF.

Consecutive patients with PsAF who underwent first-time radiofrequency catheter ablation (RFCA) were included. To assess the contribution of PVs to the underlying mechanism of AF, only patients who underwent a wide antral PVI-only approach, without adjunctive substrate-based or non-PV trigger ablation, were included. The mechanism of PsAF was classified as follows: (a) *Likely PV-dependent*, in the absence of AF or atrial flutter recurrence after stand-alone PVI and at least 18 months of follow-up (FU) off-antiarrhythmic drugs (AAD) (“PVI_responders_ group”), (b) *Definitely/likely PV-independent* (“PV_non−responders_ group”) if arrhythmia recurred beyond the blanking period despite either: (i) absence of any PV reconnection at reintervention or (ii) at least two interventions limited to PV reisolation. For patients not fulfilling the above criteria, PVI response was considered *indeterminate* (e.g. shorter FU off-AAD, additional ablation strategies beyond PVI at redo intervention, recurrence of AF after index PVI without reintervention, etc.). Details of the mapping protocol and RFCA procedure have been reported [1]. Briefly, all patients underwent pre-ablation assessment of the circumferential extent of PV connection with a 22-pole variable Lasso^®^ circular catheter (Biosense Webster, Diamond Bar, CA) placed near the ostium of each PV, but distally enough to record clearly discernible PV potentials on the circular mapping catheter electrograms. The extent of PV connection was graded by the operator in tenths of PV circumference for each vein, based on the number of bipoles recording distinct PV potentials (Fig. 1A). A mean grading of PV connections was calculated for each patient. The study was approved by the institutional ethical review board and patients provided written informed consent.

Univariate predictors of PVI success were further assessed with a multivariable logistic regression model calculating adjusted odds ratios (OR). Given the lower female representation, the sex–outcome association was assessed using a permutation test (1,000 iterations). Statistical significance was considered for *p* < 0.05.

Among the 148 included patients (mean age 64 ± 9.7 years, 73% male, median CHA_2_DS_2_Vasc score 2, mean duration of the longest continuous AF episode 7.3 ± 7 months, mean FU 39.7 ± 17.7 months), 64 patients were classified in the PVI_responders_ group and 28 in the PVI_non−responders_ group. The remaining 56 patients had an *indeterminate* response to PVI. Compared to the PVI_non−responders_ group, the PVI_responders_ group had a significantly greater circumferential extent of PV connection (68% vs. 58%, *p* = 0.0058) and a shorter duration of uninterrupted AF (mean 6.1 ± 5.3 vs. 10.3 ± 9.9 months, *p* = 0.0347). Among other clinical variables, the proportion of women was significantly higher in the PVI_non−responders_ group compared to the PVI_responders_ group (12/28, 43% vs. 8/64, 13%; *P* = 0.002; permutation test *P* = 0.005) (Fig. 1B, D). Multivariable logistic regression analysis identified one independent marker of the likely mechanism of arrhythmogenesis for each group: the extent of PV connection (OR 1.026; 95% CI: 1.000-1.054; *P* = 0.0489) was a marker of a PV-dependent mechanism, while female sex (OR 3.919; 95% CI 1.287–12.38; *P* = 0.0169) predicted a mechanism independent of PV. Uninterrupted AF duration was less predictive of a PV-independent mechanism (OR 0.9359; 95% CI 0.8629–1.005; *P* = 0.0892) (Fig. 1C).

Based on the outcome of a stand-alone PVI ablation strategy, this study demonstrates that the extent of PV electrical connection and sex are key independent predictors of the mechanistic role of PVs in PsAF. This finding stands out as both factors are not routinely considered to stratify the contribution of PVs compared to more established factors, such as the duration of uninterrupted AF or left atrial size. Poorly connected PVs and female sex may help identify patients less likely to respond to PVI alone who may benefit from first-line adjunctive ablation strategies.

The hypothesis underlying the study is based on the simple assumption that a higher extent of PV electrical connection provides a larger substrate for reentry (or triggered activity) underlying PV firing, thereby increasing the likelihood of success of a procedure limited to PVI. The present study supports this concept, showing that the extent of PV connection is indeed higher in patients who can actually be considered cured by PVI compared to those whose mechanism of AF is most probably independent of PVs.

Moreover, this study highlights an observation that is far less appreciated when trying to delineate which patients may require additional ablation in a tailored approach, namely the fact that female sex is an independent marker of a mechanism of PsAF that is independent of PVs. Previous studies investigating sex differences in clinical and electrophysiological characteristics are in line with these findings since female sex has been associated with since female sex has been associated with higher arrhythmia recurrence higher arrhythmia recurrence rates following PVI [2], more left atrial fibrosis/adverse remodeling, fewer PV reconnections at reintervention and more non-PV AF triggers with less arrhythmogenic PVs than men [3].

Uninterrupted AF duration is an established predictor of arrhythmia recurrence after catheter ablation for PsAF regardless of the ablation strategy. It is generally thought that early in the course of AF, (PV) triggers predominate while, as the arrhythmia becomes more long-lasting, the sustained high rates in the atrium and/or the presence of underlying heart disease induce alterations in the substrate that promote AF perpetuation (“AF begets AF”). A PV dependent mechanism of AF is therefore expected to be more often observed in shorter duration AF. This finding was also noted in our cohort but failed to reach statistical significance in multivariate analysis. This is likely explained by the relatively short duration of uninterrupted AF (7.3 ± 7.0 months). In a recent analysis of the EARNEST-PVI trial comparing PVI alone to PVI with additional linear ablation or defragmentation (PVI-plus) in PsAF, the authors showed that the outcomes of PVI-alone among patients with long-standing PsAF lasting 1-2.4 years were similar to those with PsAF lasting < 1 year. However, a positive effect of the PVI-plus strategy was observed over PVI-alone in patients with PsAF lasting longer than 2.4 years suggesting that longer AF durations are required to increase the proportion of patients with a “substrate-based” mechanism of AF [4].

The main limitation of the study is related to the assessment of the extent of electrical connection between PVs and the LA which may be dependent on the position depth of the circular catheter within the vein, and anatomical factors such as the potential overlap of bipoles in smaller diameter PVs. This potential bias was minimized by the use of a 3-D mapping system to ensure adequate positioning and deployment of the Lasso catheter. On the other hand, the selected methodology allows a simple qualitative method that may be easily implemented in routine clinical practice. Finally, the inclusion of relatively young patients with relatively short duration of uninterrupted AF restricts generalizability.

In conclusion, this study shows that the extent of circumferential PV electrical connection and female sex are the most significant independent predictors of the mechanistic role of PVs in PsAF. These parameters have both been underexplored and warrant further validation. They should be considered in future studies aiming to delineate in a tailored approach which patients may benefit most from first-line adjunctive ablation strategy beyond PVI.

**Fig. 1:**
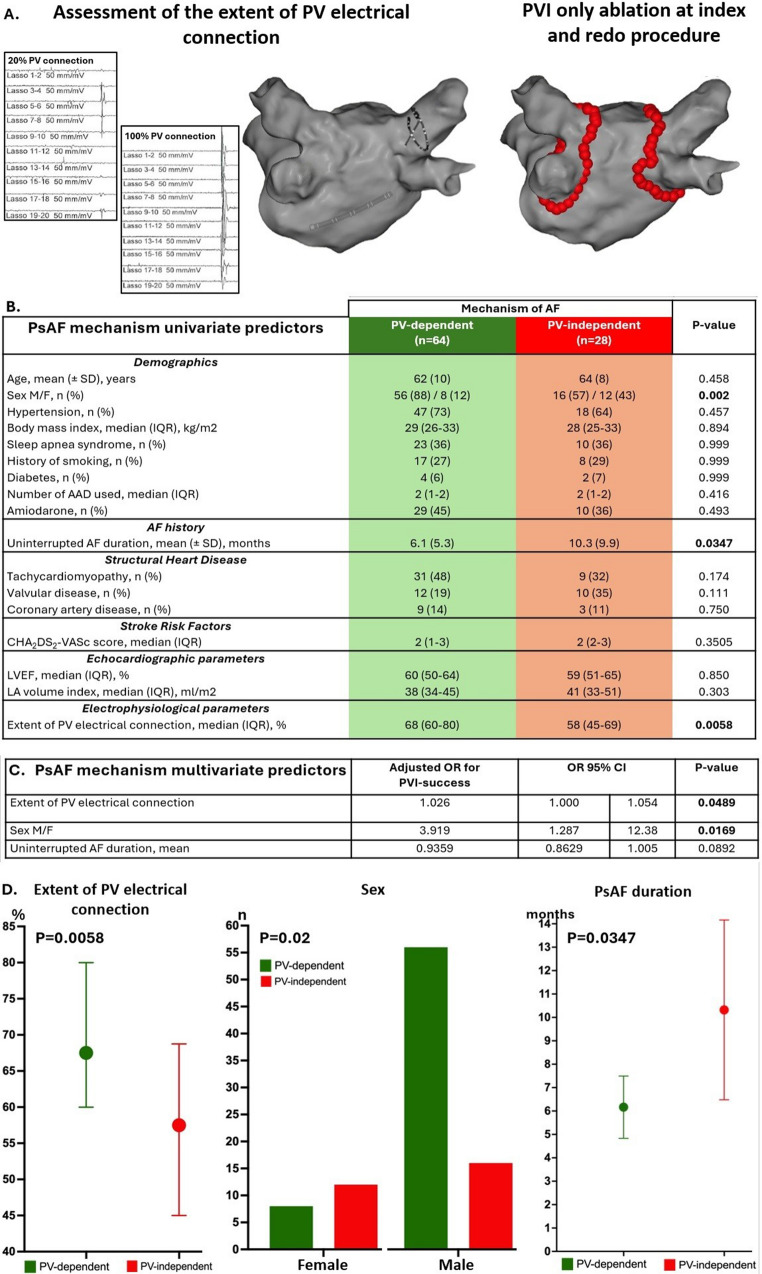
Illustration of the study design including tracings of the assessment of the extent of PV electrical connection followed by a PVI-only catheter ablation strategy (**A**). Univariate (**B-D**) and multivariate (**C**) predictors of the mechanistic role of PV in PsAF. PVI indicates pulmonary vein isolation; PsAF, persistent atrial fibrillation; F/M, female/male; AAD, antiarrhythmic drug; LVEF, left ventricular ejection fraction

## Data Availability

The data that support the findings of this study are available from the corresponding author upon reasonable request.
